# Supplementary material for: Modelling Skylarks (Alauda arvensis) to Predict Impacts of Changes in Land Management and Policy: Development and Testing of an Agent-Based Model
Source: PLoS One. 2013 Jun 6;8(6):e65803. doi: 10.1371/journal.pone.0065803 (PMC3675089; doi:10.1371/journal.pone.0065803)
Supplement: Supporting Information S4 — The skylark ODdox as a zipped archive. (ZIP) [file pone.0065803.s004.zip › Skylark_ODdox/class_movement_map.html]

ALMaSS Skylark ODdox: MovementMap Class Reference


ALMaSS Skylark ODdox
 2.0


- Main Page
- Related Pages
- Classes
- Files

- Class List
- Class Index
- Class Hierarchy
- Class Members

Public Member Functions |
Public Attributes |
Protected Member Functions |
Protected Attributes

MovementMap Class Reference

Movement maps are used for rapid computing of animal movement.
More...

`#include <MovementMap.h>`

List of all members.

|  |  |
| --- | --- |
| Public Member Functions | |
| int | GetMapValue (unsigned x, unsigned y) |
|  | MovementMap (Landscape \*L, int spref) |
| void | SetMapValue (unsigned x, unsigned y, unsigned value) |
| void | SetMapValue0 (unsigned x, unsigned y) |
| void | SetMapValue1 (unsigned x, unsigned y) |
| void | SetMapValue2 (unsigned x, unsigned y) |
| void | SetMapValue3 (unsigned x, unsigned y) |
|  | ~MovementMap () |

|  |  |
| --- | --- |
| Public Attributes | |
| uint32 \* | m\_TheMap |
| uint32 | maxx |
| uint32 | maxy |

|  |  |
| --- | --- |
| Protected Member Functions | |
| void | Init (int spref) |

|  |  |
| --- | --- |
| Protected Attributes | |
| Landscape \* | m\_ALandscape |

---

## Detailed Description

Movement maps are used for rapid computing of animal movement.

---

## Constructor & Destructor Documentation

|  |  |  |  |
| --- | --- | --- | --- |
| MovementMap::MovementMap | ( | Landscape \* | *L*, |
|  |  | int | *spref* |
|  | ) |  |  |

References Init(), m\_ALandscape, m\_TheMap, maxx, maxy, Landscape::SupplySimAreaHeight(), and Landscape::SupplySimAreaWidth().

{

m\_ALandscape = L;

// must make sure that we have whole words

maxx=(m\_ALandscape->SupplySimAreaWidth()+15)/16;

maxy=m\_ALandscape->SupplySimAreaHeight();

m\_TheMap = new uint32[ maxx\*maxy ];

Init(spref);

}

|  |  |  |  |  |
| --- | --- | --- | --- | --- |
| MovementMap::~MovementMap | ( |  | ) |  |

References m\_TheMap.

{

delete m\_TheMap;

}

---

## Member Function Documentation

|  |  |  |  |
| --- | --- | --- | --- |
| int MovementMap::GetMapValue | ( | unsigned | *x*, |
|  |  | unsigned | *y* |
|  | ) |  |  |

References m\_TheMap, and maxx.

{

uint32 theBit=x & 15;

// Multiply by two

theBit=theBit<<1;

// divide by 16

uint32 index= (x>>4)+(maxx\*y);

return ((m\_TheMap[index] >> theBit) & 0x03); // 0-3

}

|  |  |  |  |  |  |
| --- | --- | --- | --- | --- | --- |
| void MovementMap::Init | ( | int | *spref* | ) |  |

protected

References g\_msg, m\_ALandscape, SetMapValue(), Landscape::SupplyElementType(), Landscape::SupplySimAreaHeight(), Landscape::SupplySimAreaWidth(), tole\_ActivePit, tole\_AmenityGrass, tole\_BareRock, tole\_BeetleBank, tole\_Building, tole\_BuiltUpWithParkland, tole\_Coast, tole\_ConiferousForest, tole\_Copse, tole\_DeciduousForest, tole\_Field, tole\_FieldBoundary, tole\_Foobar, tole\_Freshwater, tole\_Garden, tole\_Heath, tole\_HedgeBank, tole\_Hedges, tole\_LargeRoad, tole\_Marsh, tole\_MixedForest, tole\_NaturalGrass, tole\_Orchard, tole\_OrchardBand, tole\_OrchardGrass, tole\_Parkland, tole\_PermanentSetaside, tole\_PermPasture, tole\_PermPastureLowYield, tole\_PermPastureTussocky, tole\_PitDisused, tole\_Railway, tole\_River, tole\_RiversidePlants, tole\_RiversideTrees, tole\_RoadsideVerge, tole\_RuralResidential, tole\_Saltwater, tole\_SandDune, tole\_Scrub, tole\_SmallRoad, tole\_StoneWall, tole\_Suburban, tole\_Track, tole\_UnsprayedFieldMargin, tole\_Urban, tole\_UrbanNoVeg, tole\_UrbanPark, tole\_YoungForest, MapErrorMsg::Warn(), and WARN\_FILE.

Referenced by MovementMap().

{

char error\_num[20];

for (int y=0; y<m\_ALandscape->SupplySimAreaHeight(); y++)

{

for (int x=0; x<m\_ALandscape->SupplySimAreaWidth(); x++)

{

TTypesOfLandscapeElement tole;

tole=m\_ALandscape->SupplyElementType(x,y);

uint32 colour=0;

if (spref==0)

{

// Beetle

switch (tole)

{

case tole\_Hedges: // 130

case tole\_RoadsideVerge: // 13

case tole\_FieldBoundary: // 160

case tole\_HedgeBank:

case tole\_BeetleBank:

colour=1;

break;

case tole\_Marsh: // 95

case tole\_Scrub: // 70

case tole\_Railway: // 118

case tole\_PermanentSetaside: // 33

case tole\_RiversidePlants: // 98

case tole\_PitDisused: // 75

case tole\_Garden: //11

case tole\_Track: // 123

case tole\_SmallRoad: // 122

case tole\_LargeRoad: // 121

colour=2;

break;

case tole\_Field: // 20 & 30

case tole\_UnsprayedFieldMargin :

case tole\_PermPasture: // 35

case tole\_PermPastureLowYield: // 35

case tole\_PermPastureTussocky: // 26

case tole\_Heath:

case tole\_NaturalGrass: // 110

case tole\_AmenityGrass:

case tole\_Parkland:

case tole\_Orchard: //55

case tole\_OrchardBand: //55

case tole\_OrchardGrass: //55

colour=0;

break;

case tole\_RiversideTrees: // 97

case tole\_DeciduousForest: // 40

case tole\_MixedForest: // 60

case tole\_ConiferousForest: // 50

case tole\_YoungForest: //55

case tole\_StoneWall: // 15

case tole\_Building: // 5

case tole\_Urban: // 10

case tole\_ActivePit: // 115

case tole\_Freshwater: // 90

case tole\_River: // 96

case tole\_Saltwater: // 80

case tole\_Coast: // 100

case tole\_BareRock: // 59

case tole\_UrbanNoVeg:

case tole\_UrbanPark:

case tole\_Suburban:

case tole\_RuralResidential:

case tole\_BuiltUpWithParkland:

case tole\_SandDune:

case tole\_Copse:

colour=3;

break;

case tole\_Foobar: // 999 !! type unknown - should not happen

default:

sprintf( error\_num, "%d", tole );

g\_msg->Warn( WARN\_FILE,

"MovementMap::Init(): Unknown landscape element type:",

error\_num );

exit( 1 );

}

}

else if (spref==1) { // Partridge

switch (tole)

{

case tole\_Hedges: // 130

colour=3;

break;

case tole\_PermanentSetaside: // 33

colour=3;

break;

case tole\_RoadsideVerge: // 13

colour=3;

break;

case tole\_Railway: // 118

colour=2;

break;

case tole\_FieldBoundary: // 160

case tole\_HedgeBank:

case tole\_BeetleBank:

case tole\_UnsprayedFieldMargin: // 31

colour=3;

break;

case tole\_Marsh: // 95

colour=1;

break;

case tole\_Scrub: // 70

colour=2;

break;

case tole\_Field: // 20 & 30

colour=2;

break;

case tole\_PermPasture: // 35

colour=2;

break;

case tole\_NaturalGrass: // 110

colour=2;

break;

case tole\_RiversidePlants: // 98

colour=2;

break;

case tole\_PitDisused: // 75

colour=1;

break;

case tole\_RiversideTrees: // 97

colour=2;

break;

case tole\_DeciduousForest: // 40

colour=0;

break;

case tole\_MixedForest: // 60

colour=0;

break;

case tole\_ConiferousForest: // 50

colour=0;

break;

case tole\_YoungForest: //55

colour=2;

break;

case tole\_StoneWall: // 15

colour=0;

break;

case tole\_Garden: //11

colour=1;

break;

case tole\_Track: // 123

colour=1;

break;

case tole\_SmallRoad: // 122

colour=1;

break;

case tole\_LargeRoad: // 121

colour=1;

break;

case tole\_Building: // 5

colour=0;

break;

case tole\_Urban: // 10

colour=0;

break;

case tole\_ActivePit: // 115

colour=0;

break;

case tole\_Freshwater: // 90

colour=0;

break;

case tole\_River: // 96

colour=0;

break;

case tole\_Saltwater: // 80

colour=0;

break;

case tole\_Coast: // 100

colour=0;

break;

case tole\_BareRock: // 59

case tole\_AmenityGrass:

case tole\_Parkland:

case tole\_UrbanNoVeg:

case tole\_UrbanPark:

case tole\_Suburban:

case tole\_RuralResidential:

case tole\_BuiltUpWithParkland:

case tole\_SandDune:

case tole\_Copse:

colour=0;

break;

case tole\_Foobar: // 999 !! type unknown - should not happen

default:

sprintf( error\_num, "%d", tole );

g\_msg->Warn( WARN\_FILE,

"MovementMap::Init(): Unknown landscape element type:",

error\_num );

exit( 1 );

}

} // End partridge

else if (spref==2)

{ // Spider

switch ( tole )

{

case tole\_Hedges:

case tole\_HedgeBank:

case tole\_BeetleBank:

case tole\_Marsh:

case tole\_PermanentSetaside:

case tole\_NaturalGrass:

case tole\_RiversidePlants:

case tole\_RiversideTrees:

case tole\_DeciduousForest:

case tole\_MixedForest:

case tole\_ConiferousForest:

case tole\_YoungForest:

case tole\_StoneWall:

case tole\_Building:

case tole\_Urban:

case tole\_ActivePit:

case tole\_PitDisused:

case tole\_Scrub:

case tole\_Track:

case tole\_SmallRoad:

case tole\_LargeRoad:

case tole\_BareRock:

case tole\_UrbanNoVeg:

case tole\_SandDune:

case tole\_Copse:

colour=2;

break;

case tole\_PermPasture: //moved from case 2 to case 3

case tole\_PermPastureLowYield:

case tole\_PermPastureTussocky: //moved from case 2 to case 3

case tole\_RoadsideVerge:

case tole\_Railway:

case tole\_FieldBoundary:

case tole\_Garden:

case tole\_Coast:

case tole\_Heath:

case tole\_AmenityGrass:

case tole\_Parkland:

case tole\_BuiltUpWithParkland:

case tole\_UrbanPark:

case tole\_Suburban:

case tole\_RuralResidential:

colour=3;

break;

case tole\_Freshwater:

case tole\_River:

case tole\_Saltwater:

colour=0;

break;

case tole\_Field: // Special case

case tole\_UnsprayedFieldMargin: // Special case

colour=1;

break;

default:

sprintf( error\_num, "%d", tole );

g\_msg->Warn( WARN\_FILE,

"MovementMap::Init(): Unknown landscape element type:",

error\_num );

exit( 1 );

}// End spider

}

SetMapValue(x,y,colour);

}

}

}

|  |  |  |  |
| --- | --- | --- | --- |
| void MovementMap::SetMapValue | ( | unsigned | *x*, |
|  |  | unsigned | *y*, |
|  |  | unsigned | *value* |
|  | ) |  |  |

References m\_TheMap, and maxx.

Referenced by Init().

{

uint32 theBit= x & 15; // Identify the bit within 16 possibilities

// Multiply by two

theBit=theBit<<1; // Double it because we use two bits per location

// divide by 16

// Calculate index:

uint32 index= (x>>4)+(y\*maxx); // maxx is the number of uints per row + x/16 because there are 16 locations per uint

value &= 0x03;

uint32 NewVal = m\_TheMap[index] & ~( 0x03 << theBit ); // Make a hole two bits wide at the right location

m\_TheMap[index] = NewVal | (value << theBit); // Fill the hole

}

|  |  |  |  |
| --- | --- | --- | --- |
| void MovementMap::SetMapValue0 | ( | unsigned | *x*, |
|  |  | unsigned | *y* |
|  | ) |  |  |

References m\_TheMap, and maxx.

{

uint32 theBit=x & 15;

theBit=theBit<<1; // Multiply by two

// divide by 16

uint32 index= (x>>4)+(y\*maxx);

m\_TheMap[index] &= ~(0x03<< theBit);

}

|  |  |  |  |
| --- | --- | --- | --- |
| void MovementMap::SetMapValue1 | ( | unsigned | *x*, |
|  |  | unsigned | *y* |
|  | ) |  |  |

References m\_TheMap, and maxx.

{

uint32 theBit=x & 15;

theBit=theBit<<1; // Multiply by two

// divide by 16

uint32 index= (x>>4)+(maxx\*y);

m\_TheMap[index] &= ~(0x03 << theBit);

m\_TheMap[index] |= (0x01 << theBit);

}

|  |  |  |  |
| --- | --- | --- | --- |
| void MovementMap::SetMapValue2 | ( | unsigned | *x*, |
|  |  | unsigned | *y* |
|  | ) |  |  |

References m\_TheMap, and maxx.

{

uint32 theBit=x & 15;

// Multiply by two

theBit=theBit<<1;

// divide by 16

uint32 index= (x>>4)+(maxx\*y);

m\_TheMap[index] &= ~(0x03 << theBit);

m\_TheMap[index] |= (0x02 << theBit);

}

|  |  |  |  |
| --- | --- | --- | --- |
| void MovementMap::SetMapValue3 | ( | unsigned | *x*, |
|  |  | unsigned | *y* |
|  | ) |  |  |

References m\_TheMap, and maxx.

{

uint32 theBit=x & 15;

// Multiply by two

theBit=theBit<<1;

// divide by 16

uint32 index= (x>>4)+(maxx\*y);

m\_TheMap[index] |= (0x03 << theBit);

}

---

## Member Data Documentation

Landscape\* MovementMap::m\_ALandscape

protected

Referenced by Init(), and MovementMap().

uint32\* MovementMap::m\_TheMap

Referenced by GetMapValue(), MovementMap(), SetMapValue(), SetMapValue0(), SetMapValue1(), SetMapValue2(), SetMapValue3(), and ~MovementMap().

uint32 MovementMap::maxx

Referenced by GetMapValue(), MovementMap(), SetMapValue(), SetMapValue0(), SetMapValue1(), SetMapValue2(), and SetMapValue3().

uint32 MovementMap::maxy

Referenced by MovementMap().

---

The documentation for this class was generated from the following files:

- MovementMap.h
- MovementMap.cpp


- MovementMap
- Generated on Thu Jan 10 2013 13:15:36 for ALMaSS Skylark ODdox by
   1.8.1.1
